# Behaviors Related to Psychiatric Disorders and Pain Perception in C57BL/6J Mice During Different Phases of Estrous Cycle

**DOI:** 10.3389/fnins.2021.650793

**Published:** 2021-04-06

**Authors:** Weinan Zhao, Qing Li, Yu Ma, Zhiyong Wang, Bingqian Fan, Xiaojing Zhai, Mengfan Hu, Qing Wang, Moruo Zhang, Chunyan Zhang, Yixue Qin, Sha Sha, Zhonghao Gan, Fan Ye, Yihan Xia, Guangchao Zhang, Li Yang, Shiya Zou, Zheng Xu, Sunhui Xia, Yumei Yu, Mannan Abdul, Jun-Xia Yang, Jun-Li Cao, Fang Zhou, Hongxing Zhang

**Affiliations:** ^1^Jiangsu Province Key Laboratory of Anesthesiology, Xuzhou Medical University, Xuzhou, China; ^2^Jiangsu Province Key Laboratory of Anesthesia and Analgesia Application Technology, Xuzhou Medical University, Xuzhou, China; ^3^NMPA Key Laboratory for Research and Evaluation of Narcotic and Psychotropic Drugs, Xuzhou Medical University, Xuzhou, China; ^4^School of Nursing, Xuzhou Medical University, Xuzhou, China; ^5^Department of Nursing, The Affiliated Hospital of Xuzhou Medical University, Xuzhou, China; ^6^Department of Intensive Care Medicine, The Affiliated Hospital of Xuzhou Medical University, Xuzhou, China; ^7^School of Anesthesiology, Xuzhou Medical University, Xuzhou, China; ^8^The First Medical College, Xuzhou Medical University, Xuzhou, China; ^9^Department of Anesthesiology, The Affiliated Hospital of Xuzhou Medical University, Xuzhou, China

**Keywords:** estrous cycle, female mice, psychiatry, pain, behavior

## Abstract

Robust sex difference among humans regarding psychiatry- and pain-related behaviors is being researched; however, the use of female mice in preclinical research is relatively rare due to an unchecked potential behavioral variation over the estrous cycle. In the present study, a battery of psychiatry- and pain-related behaviors are examined under physiological condition in female C57BL/6J mice over different estrous cycle phases: proestrus, estrous, metestrous, diestrous. Our behavioral results reveal that there is no significant difference over different phases of the estrous cycle in social interaction test, sucrose preference test, tail suspension test, open field test, marble burying test, novelty-suppressed feeding test, Hargreaves thermal pain test, and Von Frey mechanical pain test. These findings implicate those psychiatry- and pain-related behaviors in normal female C57BL/6J mice appear to be relatively consistent throughout the estrous cycle; the estrous cycle might not be a main contributor to female C57BL/6J mice’s variability of behaviors.

## Introduction

Animal models and behavioral tests have been used in experimental research for a long time to increase human knowledge and contribute to finding solutions to the biological and biomedical questions in ways that would be impossible in human beings (Krishnan et al., 2007; Jones et al., 2011; Esquerda-Canals et al., 2017). A dilemma in animal research is that almost all animal models and behavior tests were established and performed in male subjects (Will et al., 2017); however, diseases/symptoms, such as depression and pain, occurred more frequently in women and manifested differently in men (Kornstein et al., 2000; Calipari et al., 2017; GBD 2016 Disease and Injury Incidence and Prevalence Collaborators, 2017; Kuehner, 2017; Li and Graham, 2017). Lacking mechanistic underpinning of diseases/symptoms in female subjects might be one reason accounting for the unsatisfactory progress of current preclinical research-based translational therapeutics (Wittchen et al., 2002; Steiner et al., 2003; Jang and Elfenbein, 2019). In this case, the National Institutes of Health (NIH) of the United States raised concerns, in 2014, regarding an over-reliance on male subjects in preclinical researches, which aimed to encourage researchers to balance sex in their following cell and animal studies and grant applications (Clayton and Collins, 2014). The naturally occurring estrous cycle has been considered a significant contributor to the variation of behavioral phenotypes in female animals, leading to a serious consideration of estrous cycle effects in future studies. Early studies conducted few decades ago observed the positive impact of the estrous cycle on animal behaviors in different behavioral paradigms (Wang, 1923; Burke and Broadhurst, 1966; Guttman et al., 1975), such as measurements in the revolving wheel test and open field test (Wang, 1923; Burke and Broadhurst, 1966). The estrous cycle is also considered to play a contributing role in affecting the neuronal morphology, network activity, and function modulation in rodents (Maswood et al., 1999; Perez et al., 2014; Lisofsky et al., 2015; Richard et al., 2017), and recently, this phenomenon has re-gained increasing attraction following NIH’s announcement. In the field of psychiatry, the first batch of studies using female subjects to establish the mouse model of depression came out sequentially, two of which are chronic social defeat paradigms established on the basis of artificially induced aggression of CD1 mice to the C57BL6J female mice and the other is the vicarious social defeat model (Bourke and Neigh, 2012; Takahashi et al., 2017; Harris et al., 2018; Iñiguez et al., 2018). All of these studies have taken the effect of the estrous cycle on the behavioral outputs observed in consideration (Bourke and Neigh, 2012; Takahashi et al., 2017; Harris et al., 2018; Iñiguez et al., 2018), though none of which reported sufficient effect of estrous cycle to significantly change the performance in social interaction test. On the contrary, social interaction behavior was found to be influenced by estrous cycle in a methylazoxymethanol acetate model of schizophrenia in female rats (Perez et al., 2019). Divergent behavioral outcomes were possibly due to the difference of species, behavioral paradigms, and technical details, which made the effects of estrous cycle on animal behaviors vague and unclear. The contribution of natural estrous cycle stages to animal behaviors is still an open question and needs further investigations for studies in female animals, especially in those widely used ones.

In the present study, based on our research interest, we plan to observe psychiatry- and pain-related behavioral outputs in normal female C57BL/6J mice over different stages of estrous cycle with a battery of widely used experimental paradigms: Social interaction test, sucrose preference test, tail suspension test, open field test, marble burying test, novel environment feeding test, Hargreaves thermal test, and Von Frey mechanical test.

## Materials and Methods

### Materials

#### Animals

C57BL/6J female mice (7–8 weeks) employed in the present study were provided by Jackson Laboratory (Shanghai) through the Experimental Animal Center of Xuzhou Medical University. Before experimental tests, mice were group-housed and maintained on a 12-h light/dark cycle with food and water available *ad libitum*. All experimental protocols were approved and in accordance with the Animal Care and Use Committee of Xuzhou Medical College (Xuzhou, Jiangsu Province, China). To reduce the number of animals used in the study, some of the mice were reused in different experimental tests. The paw withdrawal latencies (PWLs) and 50% paw withdrawal threshold (50% PWTs) were tested in the same cohort of animals on the same day. The open field test and social interaction test were performed in the same cohort of animals with an interval of 7 days, to allow the animals to recover from the previous experiment’s stress. The marble burying test and novelty-suppressed feeding test (NSFT) were performed in the same cohort of animals with an interval of 7 days. To avoid the impact of tests with severe stress on animal behaviors or neuronal activity, two independent cohorts of animals were used to perform tail suspension test or sucrose preference test, respectively.

### Identification of the Estrous Cycle

The estrous cycle stage was determined with vaginal smear after behavior test as described previously by McLean et al. (2012). To this end, a micropipette with ∼100 μl sterile autoclaved double distilled water (ddH2O) was used to collect vaginal cells by gently expelling a quarter to half of the volume of water at the opening of the vaginal canal. This procedure was repeated for four–five times to collect a sufficient number of cells. The liquid was then transferred onto a glass slide and the smear was allowed to dry at room temperature completely. The slides were subjected to staining with 0.1% crystal violet (for 1 min followed by two times of wash with ddH_2_O). According to the relative ratio of vaginal cell types under light microscopy, the estrous cycle was divided into four phases: proestrus, estrous, metestrous, and diestrous. Briefly, proestrus smear contains only nucleated and cornified cells; metestrous smear contains exclusively cornified cells and an excessive number of leukocytes with relatively few nucleated and cornified cells; and during the diestrous phase, relatively less abundant leukocytes were accompanied by few nucleated and cornified cells. The vaginal cells of each mouse were collected immediately after behavior tests to identify the phase of estrous cycle.

### Open Field Test

The open field test (OFT) was performed as we previously reported (Liu et al., 2013; Cho et al., 2017), except for minor modifications in the facility size and test time. Mice were individually placed in a white plastic open-field apparatus (40 cm × 40 cm × 50 cm), which was divided into 3 × 3 subareas as one center area, four corner areas, and four side areas, illuminated by a 30-W white fluorescent light 2 m overhead. During the test, mice were placed in the center of the open apparatus from the same direction each time. After 2 min of adaptation, the total locomotor activity (time spent in different areas and distance traveled) of each animal was recorded automatically with the ANY-maze tracking system for 3 min.

### Sucrose Preference Test

Sucrose preference test (SPT) was performed as we previously reported (Wu et al., 2020). Briefly, mice were individually housed to habituate drinking water with two bottles fitted with ball-point sippers for 2 days. On the testing day, the liquid in one of the bottles with 1% sucrose and both bottles were weighed. Twelve hours later, the position of the bottles was switched to avoid the development of a side preference. At the time point of 24 h, the bottles were weighed for the second time. Sucrose preference was calculated as a percentage of sucrose solution consumption (amount of sucrose solution consumed × 100/total liquid consumption)%.

### Tail Suspension Test

Tail suspension test (TST) was performed as the method described by our previous report (Yin et al., 2011). The testing mouse was fixed with a rope at a distance of 2 cm from the tail tip and hung on a shelf with its head about 15 cm from the bottom of the testing setup. The moving activity was video-tracked for 7 min, with the data from the last 5 min used for analysis. Immobility was defined as the cessation of any bodily movements. The immobility duration for each subject within the last 5 min was recorded. Five mice that displayed tail climbing were excluded from the analysis.

### Social Interaction Test

A two-stage social interaction test (SI) was performed in a square arena (40 cm × 40 cm) with artificially defined interaction zone (14 cm × 26 cm) and corner zones (10 cm × 10 cm) as we previously reported in both male and female mice (Takahashi et al., 2017; Šabanović et al., 2020). In the first test (target-absent), the experimental mouse was allowed to freely explore the arena with an empty wire mesh sleeve (10 cm × 6 cm) in the interaction zone. In the second stage of the test, the experimental mouse was reintroduced into the arena with an unfamiliar CD1 mouse (male) in the mesh sleeve. As we previously reported, the social interaction ratio (SIR) was calculated as time in interaction zone with target/time in interaction zone without target × 100 (Zhang et al., 2019b). Video-tracking software (ANY-maze, version 4.84, Stoelting Co., Wood Dale, IL, United States) was used to measure the amount of time the experimental mouse spent in the interaction zone and corner zones.

### Novelty-Suppressed Feeding Test

As we recently reported (Hodes et al., 2015), before testing, the mice fasted for 24 h with water provided *ad libitum*. The experimental device was a standard hamster cage with the bottom covered with 2-cm corncob. A single food pellet was placed on a 10-cm Petri dish, which coved a circular white filter paper (diameter: 10 cm) in the center of the hamster cage. The experimental mouse was introduced into the corner of the cage, illuminated by a 30-W white fluorescent light 2 m overhead. The latency for mice grasping the food pellet with their forepaws and biting was recorded with an artificially controlled timer. As soon as the mice began to eat or failed to eat the food pellet within 10 min, they would be immediately transferred back to their home cage.

### Paw Withdrawal Latency

Paw withdrawal latencies (PWLs) were measured with the IITC Plantar Analgesia Meter (IITC Life Science Inc., Woodland Hills, CA, United States) in a double-blinded manner as described in our previous studies (Liu et al., 2011, 2018; Zhang H. et al., 2017; Zhang S. et al., 2017). Mice were placed in transparent acrylic enclosures (10 × 10 × 20 cm) on a glass plate in a temperature-controlled and noise-free room. The mice were allowed to habituate for 1 h before the behavioral test. A heat-producing radiant light source was used to stimulate the plantar surface of the left hind paw. Time from the “light on” to a typical withdrawal or licking of the tested hind paw was recorded as paw withdrawal latency. The basal PWLs were set to 9–15 s by adjusting the radiant light intensity. To prevent tissue damage, the radiant heat illumination was automatically cut off at 25 s. The PWLs were measured for five times/time points/animal with the last three used for analysis.

### Fifty Percent Paw Withdrawal Threshold

The measurement of the mechanical paw withdrawal threshold (PWT) was adapted from and carried out with the up-down paradigm as previously described by Chaplan et al. (1994). Mice were acclimatized for 1 h in transparent acrylic enclosures (10 × 10 × 20 cm) on a wire mesh platform in a temperature-controlled and quiet room. A sequence of calibrated Von Frey filaments (0.02, 0.04, 0.07, 0.16, 0.4, 1.0, 2, and 6 g) was chosen. The measurement was initiated with the 0.16-g hair. Each hair was applied perpendicularly to the plantar surface of the left hind paw, with sufficient force to bend the filament, for about 5 s. Lifting, shaking, or licking of the paw indicated a positive response and prompted the next weaker filament. The absence of a paw withdrawal response prompted the use of the next stronger filament. This paradigm continued until a total of six measurements or until four consecutive positive or four consecutive negative responses occurred. The 50% mechanical withdrawal thresholds were calculated as 50% PWT  =  Power[10,(Xf+κδ)] in a Microsoft Excel (2010) document; Xf = value (in log units) of the final Von Frey hair used, *κ* = tabular value [see Appendix from reference (Chaplan et al., 1994)] for the pattern of positive/negative responses, and δ = mean difference (in log units) between stimuli (here, 0.411).

### Marble Burying Behavior Test

According to the method described previously (Angoa-Pérez et al., 2013), the marble burying test was performed in a standard mouse cage with 20 marbles placed on the 5-cm depth of corncob bedding in 4 × 5 grids. The mouse was allowed 30 min to freely explore and bury marbles with two 30-W white fluorescent lights 3 m overhead. Marbles were considered buried if 2/3 or more of the marble volume was submerged. The numbers of marbles buried was recorded.

### Data Analysis and Statistics

GraphPad Prism 7.0 was used for data analysis and figure generation. All data were expressed as the mean ± SEM. Behavioral results with homoscedastic datasets were compared by one-way ANOVA followed by Tukey’s test. Data that did not pass the homoscedastic test was analyzed by a nonparametric test (Kruskal–Wallis test). Sample sizes are indicated in the figure legends, and *P* < 0.05 was considered statistically significant.

## Results

### Identification of the Estrous Cycle in Female C57BL/6J Mice

**FIGURE 1:**
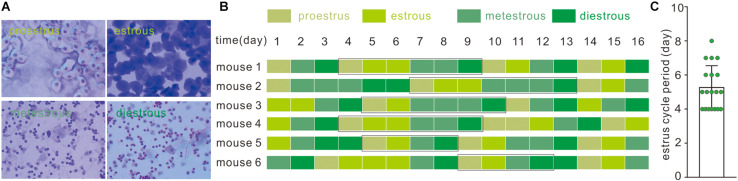
Identification of the estrous cycle phases in C57BL/6J female mice. **(A)** Vaginal cytology presenting each stage of the mouse estrous cycle. **(B)** Estrous cycles of eight C57BL/6J female mice over 16 days; black rectangle represents typical estrous cycles. **(C)** Average duration of estrous cycles of C57BL/6J female mice; data was acquired from typical estrous cycles including the above four phases in 19 mice.

Cytological components of vaginal smear determined the estrous cycle in female C57Bl/6J mice. In brief, proestrus smear contains only nucleated and cornified cells; metestrous smear contains exclusively cornified cells and an excessive number of leukocytes with relatively few nucleated and cornified cells; and during the diestrous phase, moderately less abundant leukocytes were accompanied by few nucleated and cornified cells (Figure 1A). A consecutive examination of the estrous cycle from 19 mice indicated that a typical estrous cycle lasts 4–8 days (Figures 1B,C).

### Open Field Test

**FIGURE 2:**
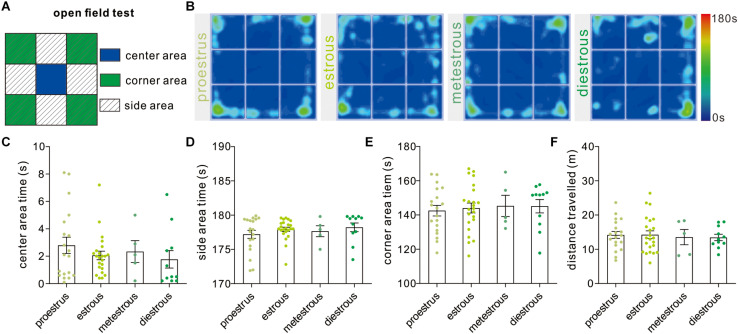
Open field test. **(A)** The schematic diagram showing center, corner, and side areas of the open field. **(B)** Representative heat maps showing time spent in each area of the open field chamber. **(C–E)** Open field analysis showing no significant difference in time spent in the center, side, and corner areas at different phases (*n* = 19, 23, 5, and 11). **(F)** Total distance traveled over the whole apparatus under different estrous cycle phases (*n* = 19, 23, 5, and 11).

The open field test developed by Calvin Hall is an experimental test extensively used to assay the level of anxiety-like behaviors and general locomotor activity (Figure 2A). In our study, the time mice spent in the center [*F*_(3,54)_ = 0.71, *P* = 0.54, one-way ANOVA], side areas [*F*_(3,54)_ = 0.70, *P* = 0.55, one-way ANOVA], and corner subareas [*F*_(3,54)_ = 0.11, *P* = 0.95, one-way ANOVA] over four estrous stages did not exhibit significant difference (Figures 2B–E). In order to evaluate the effect of the estrous cycle on animal’s locomotor activity, the total distance that mice traveled during the test was analyzed [*F*_(3,54)_ = 0.09, *P* = 0.96, one-way ANOVA], and no difference was observed between any two different stages (Figure 2F).

### Social Interaction Test

**FIGURE 3:**
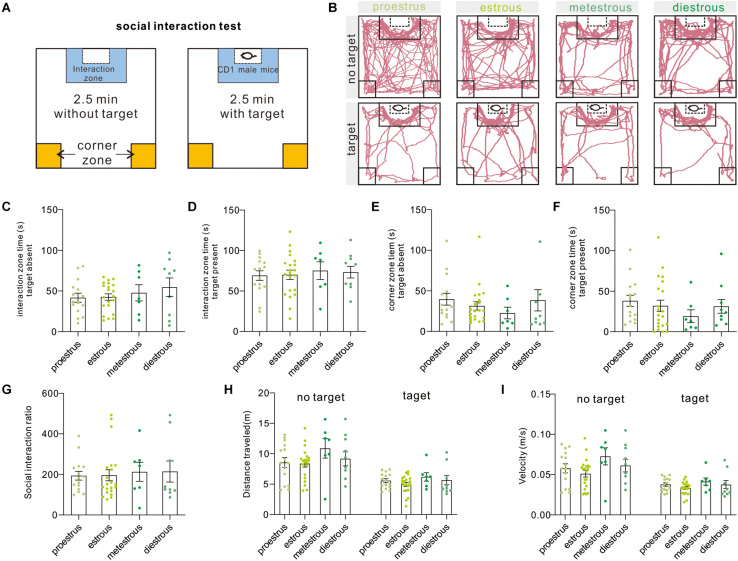
Social interaction test. **(A)** Schematic overview of the social interaction arena and behavioral test procedure. **(B)** Representative moving tracks of test mice during social interaction test at different phases of the estrous cycle. **(C,D)** Interaction zone time in the presence or absence of an unfamiliar CD1 mouse (*n* = 15, 21, 7, and 9). **(E,F)** Corner zone time in the presence or absence of an unfamiliar CD1 mouse (*n* = 15, 21, 7, and 9). **(G)** SIR of female mice at different estrous cycles (*n* = 15, 21, 7, and 9). **(H)** Distance traveled in the presence or absence of an unfamiliar CD1 mouse (*n* = 15, 21, 7, and 9). **(I)** Mean velocity in the presence or absence of an unfamiliar CD1 mouse (*n* = 15, 21, 7, and 9). SIR, social interaction ratio.

The two-stage social interaction test is an effective and widely used measurement to indicate pathology-related susceptibility to defined stress (Figure 3A), such as social avoidance in rodent models of major depressive disorder and autism disorder. In brief, decreased time in the interaction zone or increased time in corner zones indicated pathology-related social avoidance or susceptibility to defined stress. Under the present condition, we failed to observe difference in interaction zone time [target absent, *F*_(3,48)_ = 0.7291, *P* = 0.5397; target present, *F*_(3,48)_ = 0.1686, *P* = 0.9171, one-way ANOVA], corner zone time [target-absent, *F*_(3,48)_ = 0.75, *P* = 0.5277; target present, *F*_(3,48)_ = 0.6966, *P* = 0.5587, one-way ANOVA], and SIR [*F*_(3,48)_ = 0.0897, *P* = 0.9654, one-way ANOVA] over four estrous stages (Figures 3B–G). Additionally, the distance traveled [target absent, *F*_(3,48)_ = 1.29, *P* = 0.2885; target present, *F*_(3,48)_ = 1.11, *P* = 0.3542, one-way ANOVA] and mean movement velocity [target absent, *F*_(3,48)_ = 2.027, *P* = 0.1226, one-way ANOVA; target present, *H* = 3.18, *P* = 0.3647, Kruskal–Wallis test] of female mice also did not vary across phases of the estrous cycle (Figures 3H,I). Moreover, six animals were removed from the dataset because the significant outliers (SIR ≥ 600).

### Marble Burying Behavior Test

**FIGURE 4:**
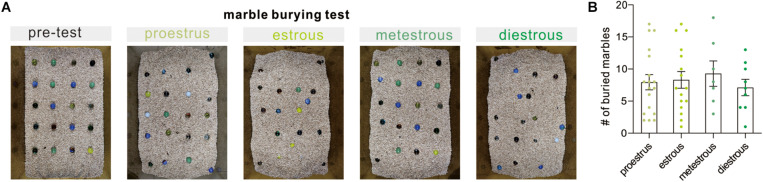
Marble burying behavior test. **(A)** Representative examples of the marble burying test arena following the test. **(B)** Quantitative data of buried marble numbers at different estrous cycles over a 30-min period (*n* = 18, 16, 7, and 9).

Marble burying is an animal behavioral test used in scientific research to depict anxiety-like behaviors, repetitive, or obsessive-compulsive disorder behavior. It is based on the observation that rats and mice will bury either harmful or harmless objects in their bedding. In the present study, our observation revealed that the number of buried marbles did not differ significantly at different phases of the cycle (*F*_(3, 46)_ = 0.2724, *P* = 0.8450, one-way ANOVA, Figure 4). The above result indicated that there is no detectable correlation between the estrous cycle and repetitive and compulsive-like behaviors in the marble burying test.

### Novelty-Suppressed Feeding Test

**FIGURE 5:**
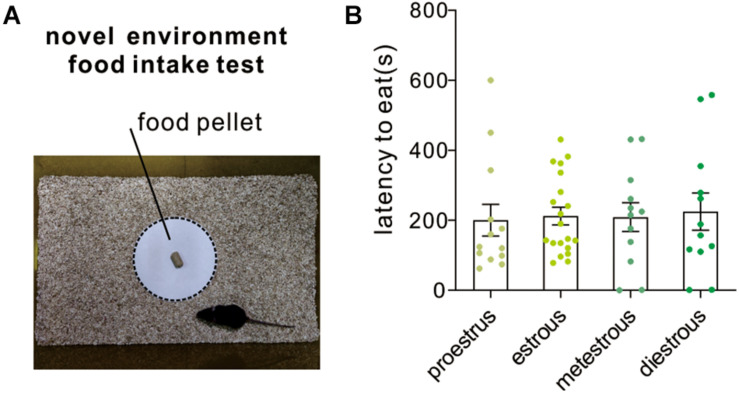
Novelty-suppressed feeding test. **(A)** An image shows the testing arena and food platform used for novel environment food intake test. **(B)** Quantitative data of feeding latency at different estrous cycles (*n* = 13, 20, 12, and 12).

Novelty-suppressed feeding test assesses the ability of the animal to resolve a conflict between a context that induces heightened anxiety-like emotionality and a drive to approach an appetitive stimulus. It is also used for assessing the efficacy of potential anxiolytic drugs. In the present study, the latency to eat the food pellet was recorded for mice under different estrous cycle stages. The anxious mice would spend a longer latency to eat. The result indicated that female mice at different stages displayed similar latency to eat [*F*_(3,53)_ = 0.05, *P* = 0.98; one-way ANOVA, Figure 5].

### Tail Suspension Test

**FIGURE 6:**
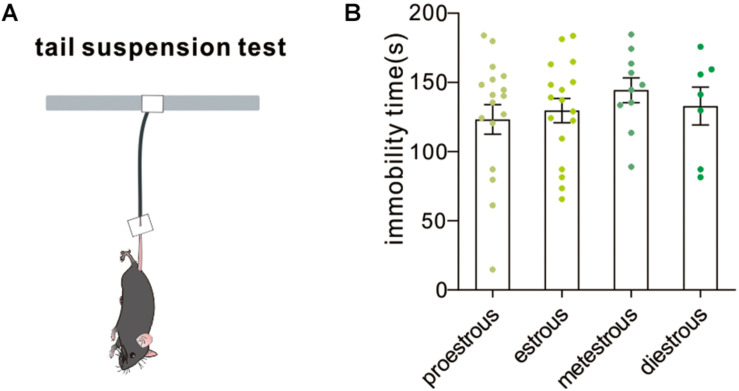
Tail suspension test. **(A)** A schematic cartoon showing tail suspension test setup. **(B)** Quantitative data of not-moving time during a 5-min tail suspension test at different estrous cycles (*n* = 17, 17, 10, and 7).

The tail suspension test is a mouse behavioral test useful in screening potential antidepressant drugs and assessing other manipulations that are expected to affect depression-related despair behaviors. Increased immobility time in the tail suspension test suggested a higher level of despair. In our experiment, no difference in immobility time among groups was observed [*F*_(3,47)_ = 0.5166, *P* = 0.6728, one-way ANOVA, Figure 6]. The result demonstrated that the estrous cycle did not affect depression-like behavior in the tail suspension test.

### Sucrose Preference

**FIGURE 7:**
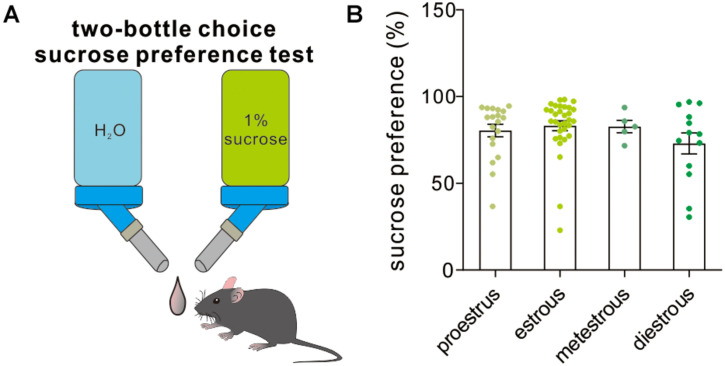
Two-bottle choice sucrose preference test. **(A)** A schematic cartoon showing two-bottle choice sucrose preference test. **(B)** Quantitative data of sucrose preference during a 12-h testing period under different estrous cycles (*n* = 19, 32, 5, and 13).

The two-bottle choice procedure for assessing sucrose preference is a useful test to investigate anhedonia (inability to feel pleasure, a core symptom of depression in humans) in laboratory rodents, particularly in stress-based models of depression. Across the four different estrous stages, the female experimental mice exhibited a similar preference to 1% sucrose solution [*F*_(3,65)_ = 1.16, *P* = 0.33, one-way ANOVA, Figure 7], which supported that the estrous cycle was not a contributor to sucrose solution-related anhedonia.

### Pain-Related Behaviors

**FIGURE 8:**
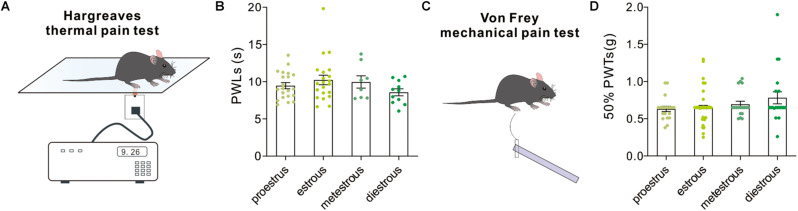
Pain-related behavioral tests. **(A)** A schematic cartoon showing the Hargreaves thermal pain test for paw withdrawal latency to a thermal stimulation. **(B)** Quantitative data of paw withdrawal latencies under different estrous cycles (*n* = 21, 21, 8, and 11). **(C)** A schematic cartoon showing the Von Frey test for paw withdrawal threshold to mechanical stimuli. **(D)** Quantitative data of 50% paw withdrawal threshold under different estrous cycles (*n* = 20, 64, 18, and 20).

Hargreaves test and Von Frey test were used to measure thermal nociceptive sensitivity and mechanical nociceptive sensitivity by quantitatively evaluating the paw withdrawal latency to noxious thermal stimulation and 50% paw withdrawal threshold to a non-noxious mechanical stimulation. In both tests, our behavioral results revealed no difference in PWLs [*F*_(3,57)_ = 1.32, *P* = 0.27, one-way ANOVA, Figures 8A,B] and 50% PWTs (*H* = 3.38, *P* = 0.3367, Kruskal–Wallis test, Figures 8C,D) in female mice among groups.

## Discussion

In this study, utilizing a battery of widely accepted behavioral tests, we examined the estrous cycle effects on a number of psychiatry- and pain-related animal behaviors in female C57BL/6J mice. Our results suggest that the behaviors described above stayed unchanged throughout different stages of the estrous cycle.

Clinical experience and scientific researches consistently agree that male and female individuals do generally differ at baseline and in response to the exposure of external stress and noxious stimuli. However, the majority of preclinical and clinical studies have been carried out in male individuals, initially due to the perception that data acquired in females under different stages of the estrous cycle would be more varied than that obtained from males. Estrogen, a hormone that fluctuates over estrous cycle, and the agonist of its receptors has also been implicated in modulating animal behaviors in a large number of studies (Crider et al., 2018; Popov et al., 2020; Qu et al., 2020; Renczés et al., 2020; Ghazvini et al., 2021; Valdés-Sustaita et al., 2021). Consistently, preclinical studies discovered a statistical difference in psychiatry- and pain-related behavioral and physiological traits (Meziane et al., 2007). For example, few strains of female mice were reported to display varied behaviors over different cycling stages in the forced swim test, social interaction test, tail suspension test, and pain-related behavioral tests (Meziane et al., 2007; Rebolledo-Solleiro and Fernández-Guasti, 2018; Chari et al., 2020). Similar behavioral fluctuations were also observed in rats in early studies (Wang, 1923; Burke and Broadhurst, 1966; Guttman et al., 1975). The effect of the estrous cycle on neuronal and molecular physiology in a few brain areas was also supported by electrophysiological studies (Calipari et al., 2017; Jaric et al., 2019). Surprisingly, few recent meta-analysis of preclinical data surprisingly indicated that the variance in data obtained from female rodents under distinct cycling stages is not different from that obtained from the males, indicating that estrous cycle is possibly not a main contributor to observed behavioral variance (Prendergast et al., 2014; Becker et al., 2016). This evidence leads to a divergence between the unchanged behavioral observations and the unstable physiology over the estrous cycle in female animals. In our view, an interpretation of this divergence is that estrous cycle-related neurobiological changes in specific brain regions is an active adaptation of the nervous system to achieve intrinsic homeostasis and maintain the stability of animal behaviors, which is essential for the survival of species (Zhang et al., 2019a).

Behavioral response to external stimuli under pathological states is more complicated than that under the physiological state. For example, following a repeated social stress model of depression, a subpopulation of mice displayed social avoidance to a novel social target in a social interaction test (susceptibility). Simultaneously, the rest remained in a normal-like social interaction behavioral phenotype (resilience) (Cao et al., 2010; Friedman et al., 2014, 2016; Walsh et al., 2014; Bagot et al., 2015; Zhang et al., 2019b). Similarly, following exposure to repeated tail shocks in a restrainer, half of the rats developed learned helplessness to later shock in a shuttle-box test, whereas the rest of the subpopulation exhibited a similar behavioral phenotype with stress-naive controls, which could also be seen as susceptibility and resilience to stress (Sartorius et al., 2003; Taneja et al., 2011). The behavioral phenotypes following pathological models will be more complicated when mixed with the effect of estrous cycle. Moreover, the underlying mechanisms, for example, the active homeostasis in the midbrain dopamine neurons, are even more complicated than the behavioral outcomes. In this case, it would be necessary to look at the effect of the estrous cycle on behaviors following specific animal models.

Our data obtained from the behavioral tests contrasts with the findings observed in a previous research on the female rodents. This study found a significant effect of the Whitten effect-induced estrous cycle on pain behaviors with tail-flick test and hot plate test to thermal stimulation, anxiety-related behaviors in open field test, and despair behavior in tail suspension test, in BALB/cByJ strains, but the estrous cycle of female C57BL/6J mice only affects despair behavior in the tail suspension test (Meziane et al., 2007). In contrast with the data presented by Meziane et al. (2007), utilizing Hargreaves thermal pain test, Von Frey Mechanical pain test, open field, and tail suspension setups, we did not observe any difference of behavior regarding pain over the different phases of the whole estrous cycle. This divergence of the tail suspension test possibly comes from a methodological variability between studies. Taken together, their study and ours indicated that there is a significant difference in the effect of estrous cycle on animal behaviors among mouse strains, and this effect appeared not remarkable in C57BL/6J mice no matter if the estrous cycle is artificially induced or naturally happened.

There are limitations in this study. First, to reduce the number of animals used in this study, some animals were used for two different behavioral paradigms, for example, in the Hargreaves thermal pain test and Von Frey mechanical pain test, in the open field test and social interaction test, as well as in the marble burying test and novelty suppressed feeding test. It is widely accepted that these behavioral tests generate weak, if not, no stress on the test animals, especially after a long-term recovery. However, we could not completely exclude the possibility that the first behavioral test and the virginal smear test that followed would affect the second behavioral test’s data. Second, the present study was conducted with a relatively small sample size, though acceptable for studies with male animals, affecting the data collection and interpretation. Third, behavioral data from the first 2 min in the open field test was not included in the data analysis, which might represent as an essential reflection of anxiety-like behaviors.

In summary, our study has suggested that the estrous cycle is not a key effector of variance in psychiatry- and pain-related behaviors in female C57BL/6J mice, and behavioral tests blind to the estrous cycle might be acceptable in naive mice. It is also proposed that the potential interaction between estrous cycle and external stimuli may lead to complex behavioral outcomes; in line with this phenomenon, it is suggested that the future investigation should be more attentive toward the influence of estrous cycle on animal behaviors in pathological states, such as specific animal models that reflect pathological conditions of human diseases.

## Data Availability Statement

The original contributions presented in the study are included in the article/supplementary material, further inquiries can be directed to the corresponding author/s.

## Ethics Statement

The animal study was reviewed and approved by the Animal Care and Use Committee of Xuzhou Medical University.

## Author Contributions

All authors listed have made a substantial, direct and intellectual contribution to the work, and approved it for publication.

## Conflict of Interest

The authors declare that the research was conducted in the absence of any commercial or financial relationships that could be construed as a potential conflict of interest.
